# Impact of Vegan and Vegetarian Diets on Neurological Health: A Critical Review

**DOI:** 10.3390/nu17050884

**Published:** 2025-02-28

**Authors:** Vicente Javier Clemente-Suárez, Laura Redondo-Flórez, Alexandra Martín-Rodríguez, Agustín Curiel-Regueros, Alejandro Rubio-Zarapuz, José Francisco Tornero-Aguilera

**Affiliations:** 1 Faculty of Medicine, Health and Sports, Universidad Europea de Madrid, Villaviciosa de Odón, 28670 Madrid, Spain; vctxente@yahoo.es (V.J.C.-S.); sandra.martin.rodriguez8@gmail.com (A.M.-R.); curielagus@gmail.com (A.C.-R.); 2 Grupo de Investigación en Cultura, Educación y Sociedad, Universidad de la Costa, Barranquilla 080002, Colombia; 3 Department of Health Sciences, Faculty of Biomedical and Health Sciences, Universidad Europea de Madrid, Tajo Street s/n, 28670 Villaviciosa de Odon, Spain; lauraredondo_1@hotmail.com; 4 Faculty of Applied Social Sciences and Communications, UNIE University, 28015 Madrid, Spain; 5 Kos Generating Health, 45007 Toledo, Spain; doctorneroaguilera@gmail.com

**Keywords:** vegan, vegetarian, diet, inflammation, plant-based, nutrition

## Abstract

Background/Objectives: The global shift towards vegan and vegetarian diets has garnered attention for their ethical, environmental, and potential health benefits. These diets are often rich in phytonutrients and antioxidants, which have been associated with lower levels of inflammatory markers, such as C-reactive protein (CRP) and interleukin-6 (IL-6), suggesting a potential protective effect against systemic inflammation and oxidative stress. However, despite these benefits, concerns remain regarding their impact on neurological health due to the possible deficiencies of critical nutrients such as vitamin B12, DHA, EPA, and iron. This review critically evaluates the influence of these dietary patterns on neurological outcomes, emphasizing their nutritional composition, potential deficiencies, and their interplay with inflammation and oxidative stress. Methods: A systematic review of the literature published between 2010 and 2023 was conducted, focusing on studies that explore the relationship between vegan and vegetarian diets and neurological health. Key nutrients such as vitamin B12, omega-3 fatty acids, iron, and zinc were analyzed alongside antinutritional factors and their effects on the nervous system. Results: Evidence suggests that vegan and vegetarian diets, when well planned, can be rich in phytonutrients and antioxidants, which have been associated with lower levels of inflammatory markers, such as C-reactive protein (CRP) and interleukin-6 (IL-6). These findings indicate a potential role in reducing systemic inflammation and oxidative stress, both of which are linked to neurodegenerative diseases. However, deficiencies in critical nutrients such as vitamin B12, DHA, EPA, and iron have been consistently associated with an increased risk of cognitive decline, mood disturbances, and neurodegenerative disorders. Additionally, the presence of antinutritional factors like phytates and oxalates may further impair nutrient absorption, necessitating careful dietary planning and supplementation. Conclusions: While plant-based diets provide anti-inflammatory and antioxidant benefits, their neurological implications depend on nutrient adequacy. Proper planning, supplementation, and food preparation techniques are essential to mitigate risks and enhance cognitive health. Further research is needed to explore long-term neurological outcomes and optimize dietary strategies.

## 1. Introduction

The global adoption of vegan and vegetarian diets has grown exponentially, largely influenced by ethical concerns regarding animal welfare, environmental sustainability, and perceived health benefits. Interest in vegan and vegetarian diets has grown globally. The campaign Veganuary saw over 700,000 participants in 2022. Germany led Google searches on veganism in 2023, followed by Austria and the UK. Global retail sales of plant-based meat products reached USD 6.1 billion in 2022. A 2021 survey found 81% of consumers tried plant-based milk, 48% tried other dairy alternatives, 44% tried vegan meat, and 25% tried a vegan egg substitute. Searches for “vegan food near me” spiked over 5000% in 2021, and the vegan leather market is projected to reach nearly USD 90 billion by 2025 [1]. By regions, in India, estimates suggest that 20–40% of the population follows a vegetarian diet, with veganism slowly growing in urban centers. In contrast, the United Kingdom reports around 5–7% vegetarians and 2% vegans, while in the United States, approximately 5% of adults are vegetarian and 3% identify as vegan. Similarly, Germany has about 10% vegetarians and 1–2% vegans, and Australia shows roughly 10–12% adherence to vegetarian diets with 2–3% following vegan lifestyles [1].

These dietary patterns, characterized by the exclusion of meat and other animal-derived products (in the case of veganism), are frequently associated with reduced risks of chronic diseases such as cardiovascular disease, hypertension, type 2 diabetes, and obesity [1,2]. Emerging research also highlights potential advantages for gut health and immune function due to the high intake of fiber and phytonutrients prevalent in vegan and vegetarian diets [2]. Despite these benefits, the impact of these diets on neurological health remains an underexplored area of research. The brain relies heavily on nutrients such as vitamin B12, omega-3 fatty acids, iron, and zinc for optimal functioning, many of which may be less bioavailable in vegan and vegetarian diets [3]. Additionally, plant foods often contain antinutritional factors such as phytates and lectins that may impair nutrient absorption, raising questions about their long-term effects on cognitive function and overall neurological health. Understanding the nuanced relationship between plant-based dietary patterns and neurological outcomes is essential to ensure that the benefits of these diets are not overshadowed by potential deficiencies or adverse effects.

Despite the potential benefits of vegan and vegetarian diets, their impact on neurological health is a growing area of concern due to the reliance of the nervous system on specific nutrients that are either limited or less bioavailable in vegan and vegetarian diets. For instance, vitamin B12, which is predominantly found in animal-derived products, plays a crucial role in myelin synthesis and neurotransmitter production, and its deficiency is linked to neurodegenerative diseases, cognitive decline, and neuropathy [4]. Similarly, omega-3 fatty acids, particularly eicosapentaenoic acid (EPA) and docosahexaenoic acid (DHA), are vital for maintaining neuronal membrane fluidity and synaptic plasticity; yet, these fatty acids are scarce in vegan diets, with plant-based sources providing only alpha-linolenic acid (ALA), which has low conversion rates to EPA and DHA [5]. Iron and zinc, essential for neurotransmitter function and immune responses, are also less bioavailable in vegan and vegetarian diets due to the presence of phytates and polyphenols, which inhibit mineral absorption [6]. These deficiencies may have profound implications for cognitive function, mood regulation, and the risk of neurodegenerative diseases, underscoring the importance of dietary planning and supplementation in individuals adhering to these dietary patterns.

The potential risks associated with vegan and vegetarian diets are further com-pounded by the presence of antinutritional factors in plant-based foods. These compounds, which include phytates, lectins, oxalates, and tannins, are naturally occurring chemical defenses in plants designed to deter herbivory, but can interfere with human nutrient absorption and metabolism. Phytates, for instance, bind minerals like iron, zinc, and calcium, forming insoluble complexes that reduce their bioavailability [6]. Lectins, found in legumes and grains, can impair gastrointestinal function and lead to systemic inflammation when consumed in excess or not properly neutralized through cooking [7]. Oxalates, abundant in foods like spinach and rhubarb, have been associated with the formation of kidney stones and potential neurotoxic effects through calcium chelation [8]. While cooking and food preparation techniques, such as soaking, fermenting, and sprouting, can mitigate these effects, the potential cumulative impact of antinutritional factors on neurological health remains underexplored. These factors may exacerbate nutrient deficiencies already prevalent in vegan and vegetarian diets, further emphasizing the need for careful dietary management and supplementation strategies.

In addition to nutritional deficiencies and antinutritional factors, the effects of vegan and vegetarian diets on neurological health are also mediated by their impact on inflammation and oxidative stress. Vegan and vegetarian diets are generally associated with lower levels of systemic inflammation due to their high content of antioxidants and anti-inflammatory phytonutrients, such as flavonoids, carotenoids, and polyphenols [9]. These compounds scavenge free radicals, reduce oxidative stress to cellular structures, and modulate inflammatory pathways, potentially offering neuroprotective effects [10]. However, the low intake of critical anti-inflammatory nutrients like omega-3 fatty acids, especially EPA and DHA, in vegan diets can offset these benefits, as these fatty acids play a pivotal role in regulating neuroinflammation and protecting neuronal integrity [11]. Furthermore, deficiencies in vitamin B12 and iron, common in vegan and vegetarian diets, have been linked to elevated homocysteine levels, a known risk factor for oxidative stress and neurodegenerative diseases [12]. These conflicting factors highlight the complexity of the relationship between vegan and vegetarian diets and neurological health, suggesting that, while these diets may offer protective benefits against inflammation and oxidative stress, they may also introduce vulnerabilities if not adequately balanced through supplementation and strategic dietary planning. In addition to the widely studied nutritional aspects, the role of antinutrients in neurological health warrants greater attention. Antinutrients, present in many plant-based foods, can influence the bioavailability of critical nutrients and disrupt metabolic pathways related to neurological function, such as inflammation and oxidative stress. However, current research lacks a comprehensive understanding of the precise mechanisms through which these compounds affect the nervous system, limiting evidence-based recommendations for vegan and vegetarian diets. Therefore, this review aims to critically evaluate the relationship between antinutrients and neurological health, highlighting their potential detrimental and protective effects, and identifying gaps in the literature to guide future research [3,9].

To conduct this critical review, we adhered to established methodologies commonly employed in similar review articles to ensure a rigorous and comprehensive approach. Critical reviews play a pivotal role in synthesizing existing knowledge, identifying research gaps, and evaluating the quality and reliability of published studies. By integrating and analyzing findings from diverse sources, critical reviews contribute to the advancement of scientific understanding and guide future research directions. This review methodology emphasizes the importance of systematically assessing the evidence to provide a balanced and insightful perspective on the impact of vegan and vegetarian diets on neurological health. To this end, a systematic review of the literature was conducted, adhering to established guidelines for critical reviews. The search strategy included multiple academic databases, such as PubMed, Scopus, and Web of Science, using a combination of keywords and Boolean operators. Key terms included “vegan diet”, “vegetarian diet”, “neurological health”, “cognitive function”, and “nutritional deficiencies”, among others. The search focused on articles published between 1 January 2010 and 1 November 2024 to capture the most relevant and up-to-date research. However, earlier studies were included when necessary to provide foundational or contextual information. The following exclusion criteria were applied: (i) studies unrelated to vegan or vegetarian diets and their effects on neurological health, (ii) publications with methodological flaws or insufficient relevance to the review’s objectives, and (iii) non-peer-reviewed sources such as PhD dissertations, conference proceedings, abstracts, and unpublished studies. All articles meeting the inclusion criteria were assessed by the review’s seven authors, who evaluated their relevance, scientific rigor, and connection to the review’s subsections. Discrepancies in article selection or interpretation were resolved through group discussion to ensure consensus. To ensure a rigorous review, a predefined data analysis strategy was employed. The quality of the included studies was assessed using the Cochrane Risk of Bias Tool and the Newcastle–Ottawa Scale depending on the study design. These tools evaluated methodological soundness based on criteria such as selection bias, measurement accuracy, and reporting transparency. In total, 112 studies were reviewed, encompassing both observational and interventional designs. Geographically, the reviewed studies represented a global distribution, with the majority originating from North America (35%), Europe (28%), and Asia (22%). Methodologically, 60% of the studies were cross-sectional, 25% were randomized controlled trials (RCTs), and 15% were longitudinal cohort studies. Furthermore, findings from preclinical in vitro and in vivo studies were incorporated, offering relevant data on the molecular and cellular mechanisms involved in the response to nutritional deficiencies and the presence of antinutrients. This combination of clinical and experimental approaches allows for a more complete and substantiated view of the neurobiological impact of these diets. Thus, this diverse dataset provided a comprehensive foundation for examining the impact of vegan and vegetarian diets on neuro-logical health.

The objective of this systematic review is to critically evaluate the impact of vegan and vegetarian diets on neurological health by analyzing their nutritional composition, potential deficiencies, and the mechanisms through which these dietary patterns influence cognitive function, mood regulation, and the risk of neurodegenerative diseases. Thus, our review integrates results from a wide range of observational and interventional studies carried out globally, emphasizing the dual function of antinutrients in plant-based diets. This approach provides a thorough understanding of how these diets can enhance cognitive health while considering the associated risks and benefits related to nutrient adequacy.

## 2. Nutritional Composition of Vegan and Vegetarian Diets

It is essential to distinguish and compare the nutritional composition of vegan and vegetarian diets [13]. Vegan diets exclude all animal-derived products, which results in nutritional profiles characterized by lower intakes of vitamin B12, vitamin D, omega-3 fatty acids, iron, calcium, and iodine compared to both vegetarian and omnivorous diets. In contrast, vegetarian diets, which may include dairy and eggs, partially mitigate these deficiencies while still offering higher intakes of dietary fiber, magnesium, and antioxidants relative to omnivorous diets. Recent evidence indicates that these distinct nutrient profiles not only influence overall nutritional adequacy, but also have significant implications for neurological function and mental health [13].

Emerging research reveals a multifaceted interplay between these diets, gut health, inflammation, and cognitive outcomes, underscoring their potential to influence mental and neurological well-being positively or negatively depending on their composition and quality. One of the most compelling aspects of vegan and vegetarian diets is their impact on the gut–brain axis, a bidirectional communication system linking the central nervous system and the gut microbiome. High dietary fiber intake, a hallmark of vegan and vegetarian diets, supports the proliferation of beneficial gut bacteria such as *Bifidobacteria* and *Lactobacillus*. These bacteria produce short-chain fatty acids (SCFAs) like butyrate, which play a critical role in maintaining the integrity of the blood–brain barrier and reducing neuroinflammation [14]. For example, studies have shown that individuals consuming vegan and vegetarian diets exhibit higher fecal butyrate levels, correlating with improved markers of systemic inflammation and cognitive performance [15]. This gut-mediated mechanism may be particularly relevant in conditions such as Parkinson’s disease, where gut microbiome dysbiosis often precedes clinical symptoms [16].

Beyond the gut microbiome, the systemic anti-inflammatory effects of vegan and vegetarian diets further contribute to their neurological benefits. Chronic low-grade inflammation is a well-established driver of neurodegenerative diseases, including Alzheimer’s and Parkinson’s, as well as mood disorders like depression. A meta-analysis by Tansey et al. (2022) demonstrated that individuals adhering to vegan and vegetarian diets consistently show lower levels of pro-inflammatory markers such as C-reactive protein (CRP) and interleukin-6 (IL-6) [16]. This reduction in systemic inflammation is likely attributable to the high intake of antioxidant-rich foods, such as fruits, vegetables, and whole grains, which counteract oxidative stress and mitigate the effects of aging on the brain. For instance, polyphenols found in berries and green tea have been shown to inhibit the production of reactive oxygen species, a key factor in the pathogenesis of Alzheimer’s disease [17].

Another dimension of neurological health influenced by vegan and vegetarian diets is their impact on mood and mental well-being. Psychological studies have increasingly highlighted the link between dietary patterns and mental health outcomes. Vegan and vegetarian diets, due to their nutrient-dense composition, may support the production of mood-regulating neurotransmitters such as serotonin and dopamine. Tryptophan, an amino acid found in plant-based proteins like soy and pumpkin seeds, serves as a precursor for serotonin synthesis. A randomized controlled trial (RCT) by Dinu et al. (2022) found that participants who transitioned to a vegetarian diet experienced significant improvements in self-reported measures of mood and anxiety, likely due to reduced inflammation and enhanced serotonin production. However, it is essential to recognize the potential for mood disturbances in poorly planned vegan diets, particularly those lacking critical nutrients such as vitamin B12 and omega-3 fatty acids [18]. Deficiencies in these nutrients are associated with an increased risk of depression and cognitive decline, emphasizing the importance of dietary adequacy and supplementation when necessary [19].

The neuroprotective potential of vegan and vegetarian diets extends to their ability to reduce the risk of neurodegenerative diseases. Epidemiological studies suggest that individuals following vegan and vegetarian diets have a lower incidence of Alzheimer’s disease and other forms of dementia. This association is likely mediated by the high intake of dietary antioxidants and anti-inflammatory compounds, which protect against the oxidative stress and amyloid plaque formation characteristic of Alzheimer’s pathology [20]. Additionally, diets rich in whole grains and legumes have been linked to improved insulin sensitivity and glucose regulation, both of which are critical for maintaining neuronal function and reducing the risk of vascular dementia [21].

The cardiovascular benefits of vegan and vegetarian diets also play an indirect but significant role in neurological health. Improved lipid profiles, reduced blood pressure, and enhanced endothelial function collectively support cerebral perfusion, ensuring adequate oxygen and nutrient delivery to the brain [22]. For example, a cohort study by Satija et al. (2016) demonstrated that adherence to a plant-based diet was associated with a 25% reduction in the risk of stroke, a major contributor to vascular cognitive impairment. This finding highlights the interconnectedness of systemic and neurological health, illustrating how the vascular benefits of vegan and vegetarian diets extend to cognitive preservation [22].

While the benefits of vegan and vegetarian diets are evident, their potential drawbacks must also be acknowledged. Nutritional deficiencies, particularly in vitamin B12, iron, zinc, and omega-3 fatty acids, pose challenges for neurological health if not addressed through proper dietary planning. For example, vitamin B12 deficiency, which is common in vegan populations, can lead to irreversible neurological damage, including myelin sheath degeneration and cognitive impairments. A longitudinal study by Rizzo et al. (2021) found that nearly 40% of vegans who did not use B12 supplements exhibited early signs of neuropathy [23]. Similarly, the low bioavailability of non-heme iron and zinc in vegan and vegetarian diets requires strategies such as the inclusion of vitamin C-rich foods to enhance absorption [24].

Recent advances in nutrigenomics offer promising avenues for optimizing the neurological benefits of vegan and vegetarian diets. By identifying genetic polymorphisms that affect nutrient metabolism and utilization, personalized dietary recommendations can be developed to address individual needs. For instance, individuals with reduced efficiency in converting alpha-linolenic acid (ALA) to docosahexaenoic acid (DHA) may benefit from algae-based DHA supplements, while those with genetic variations affecting vitamin B12 absorption may require higher supplement doses or alternative delivery methods [25] This precision nutrition approach holds the potential to mitigate the risks associated with vegan and vegetarian diets while maximizing their benefits. A summary can be found on **Table 1**.

**Table 1:** Detailed summary of nutritional aspects of vegan and vegetarian diets and their implications for neurological health.

Nutrient/Component	Presence in Plant-Based Diets	Impact on Mental Health	References
Carbohydrates and Fiber	High presence due to the abundance of whole grains, legumes, fruits, and vegetables.	They promote prebiotic effects, support gut microbiota diversity, and help regulate glucose and lipid levels, benefiting brain function.	Craig & Mangels, 2009 [3]
Protein Intake	Adequate when combining various plant sources; however, it may lack a complete amino acid profile.	Tryptophan acts as a precursor to serotonin, essential for mood regulation.	Leitzmann, 2014 [25]
Omega-3 Fatty Acids	Low levels of EPA and DHA; ALA is found in foods such as flaxseeds, chia, and walnuts, though conversion to EPA/DHA is low.	Essential for neuronal health and cognitive function; deficiency may affect membrane fluidity and cognitive performance.	Saunders et al., 2021 [26]
Vitamin B12	Absent in non-fortified plant foods; present in vegetarian diets (through dairy and eggs), but insufficient in vegans without fortification or supplements.	Crucial for DNA synthesis, myelin formation, and neurotransmitter metabolism; deficiency can lead to cognitive decline and mood disorders.	Pawlak et al., 2013 [27]
Iron	Found as non-heme iron, which has lower bioavailability, with absorption inhibited by phytates; absorption can be improved with vitamin C intake.	Fundamental for oxygen transport and brain metabolism; deficiency is associated with cognitive decline and anemia.	Hunt, 2003 [28]
Zinc	Available in legumes, seeds, and nuts, although absorption is reduced due to the presence of phytates.	Vital for enzymatic activity, synaptic plasticity, and neurogenesis; deficiency can affect memory, learning, and other cognitive processes.	Weaver, 2013 [29]
Calcium	May be insufficient in vegan diets without the use of fortified products (plant milks, tofu) or supplements.	Important for neuronal excitability and synaptic transmission; deficiency can affect neuronal signaling and increase neurovascular risks.	Peneau et al., 2008 [30]
Iodine	Frequently low due to the absence of sources such as fish, dairy, or iodized salt in vegan diets.	Essential for the production of thyroid hormones, which influence cognitive function and mood regulation; deficiency can lead to hypothyroidism.	Peneau et al., 2008 [30]
Selenium	Usually deficient in vegan diets; acts as a cofactor in antioxidant enzymes such as glutathione peroxidase.	Involved in neurotransmitter synthesis and mood regulation; deficiency can increase vulnerability to neurological disorders.	Peneau et al., 2008 [30]
Phytonutrients	High presence of bioactive compounds (polyphenols, flavonoids, carotenoids) from fruits, vegetables, and whole grains.	Possess anti-inflammatory and antioxidant properties, protecting against neurodegeneration and supporting cognitive function.	Middleton et al., 2000 [31]
Inflammation and Oxidative Stress	Characterized by a low intake of pro-inflammatory foods and a high intake of antioxidants, although no exact percentages are specified.	Associated with reduced levels of inflammatory markers (such as C-reactive protein), which correlates with a lower risk of neurodegenerative diseases and mood disorders.	Tonstad et al., 2013 [32]

## 3. Antinutrients in Plants

Plants, as sessile organisms, have developed an array of chemical defense mechanisms to deter herbivory, protect against pathogens, and ensure survival in various environments. These mechanisms often involve the production of secondary metabolites, which, while beneficial to plants, can pose challenges to human nutrition and health. Many of these compounds, collectively referred to as antinutrients, interfere with nutrient absorption or exert toxic effects when consumed in excessive amounts. However, understanding the dual role of these substances—both as plant defenses and potential contributors to human health—requires a nuanced examination.

### 3.1. Plant Secondary Metabolites as Antinutrients

Plants synthesize a diverse array of secondary metabolites that, while crucial for their own defense and survival, can act as antinutrients in the human diet [33]. These compounds—such as tannins, alkaloids, glucosinolates, phytates, and lectins—can interfere with nutrient absorption. For instance, tannins bind to dietary proteins and digestive enzymes, reducing protein digestibility, and phytates chelate minerals like iron, zinc, and calcium, thereby hindering their bioavailability [34]. Additionally, alkaloids and glucosinolates, present in certain vegetables, may diminish nutrient utilization when ingested in excessive amounts. Although these metabolites play important roles in plants, their presence in food necessitates careful management through processing techniques to minimize potential nutritional drawbacks [35].

### 3.2. Antinutrients in Plant-Based Foods

Antinutrients are naturally occurring compounds in plants that can impair the absorption or metabolism of vital nutrients in humans. While these compounds serve critical protective functions for plants—such as deterring herbivores and pathogens—they may present nutritional challenges when consumed in significant quantities. However, their effects can often be mitigated through food preparation and dietary strategies.

Phytates (Phytic Acid): Found abundantly in seeds, legumes, and whole grains, phytates bind essential minerals like iron, zinc, calcium, and magnesium, forming insoluble complexes that reduce their bioavailability. This poses a particular risk for individuals dependent on vegan and vegetarian diets in regions where mineral deficiencies are prevalent. However, traditional food preparation methods such as soaking, sprouting, and fermentation can degrade phytates, significantly improving mineral absorption [36]. Recent research also suggests that phytates possess antioxidant properties, contributing to cellular health and chronic disease prevention [37].

Oxalates: Present in spinach, rhubarb, and other leafy greens, oxalates form insoluble salts with calcium, potentially decreasing calcium absorption and increasing the risk of kidney stones in susceptible individuals. Nevertheless, the overall risk of oxalate-related health issues is low for most healthy individuals consuming balanced diets. Studies have also suggested that oxalates may have a protective role against certain pathogens by reducing their access to calcium [38].

Lectins: Found primarily in legumes like beans and lentils, lectins are proteins that bind carbohydrates and can disrupt intestinal function if consumed raw or inadequately cooked. Symptoms of lectin toxicity include gastrointestinal discomfort and nutrient malabsorption. Cooking legumes at high temperatures effectively deactivates lectins, making them safe for consumption and nutritionally beneficial [39].

Tannins: Found in tea, coffee, and certain fruits, tannins can inhibit non-heme iron absorption, potentially contributing to iron deficiency anemia in populations with low dietary iron intake. However, moderate tannin consumption in a well-rounded diet is unlikely to cause harm and may even provide antioxidant and antimicrobial benefits [40].

While traditionally regarded as detrimental, antinutrients play complex roles in plant biology and human health. By understanding their mechanisms and employing effective food processing techniques, it is possible to minimize their negative impacts while harnessing their potential benefits.

### 3.3. Biological Role and Processing Benefits of Plant Antinutrients

The presence of antinutrients in plants is an evolutionary trait that enhances plant fitness by deterring pests and pathogens; however, these same compounds can pose challenges for human nutrition [41]. For example, phytates, which serve as a storage form of phosphorus in seeds, inhibit the absorption of essential minerals in the human gut. Similarly, lectins, involved in cell recognition and plant defense, can disrupt gastrointestinal function if not properly neutralized. Recognizing these dual roles, various food processing methods—such as soaking, sprouting, and cooking—have been developed to reduce antinutrient levels, thereby optimizing nutrient bioavailability and maximizing the health benefits of plant-based diets [41,42].

### 3.4. Dual Role of Antinutrients in Human Health

While antinutrients are often viewed as detrimental, many of these compounds have demonstrated potential health benefits when consumed in moderate amounts.

Phytates: Although phytates reduce mineral bioavailability, they also exhibit antioxidant properties and have been linked to reduced risks of certain cancers, cardiovascular diseases, and diabetes. Their ability to chelate excess iron may prevent oxidative stress caused by free radicals [43].

Glucosinolates: The isothiocyanates derived from glucosinolates have been extensively studied for their role in activating detoxification enzymes, reducing inflammation, and inhibiting carcinogenesis. Regular consumption of cruciferous vegetables is associated with a reduced risk of several cancers, including lung, breast, and prostate cancers [44].

Lectins: While toxic in their raw form, lectins have demonstrated immunomodulatory properties in laboratory studies, suggesting a potential role in regulating immune responses. For instance, specific lectins may enhance mucosal immunity or exhibit antiviral activity [45].

Tannins: Tannins exhibit antimicrobial and antioxidant activities, contributing to the inhibition of pathogenic bacteria and the prevention of oxidative stress in the body. In traditional medicine, tannin-rich extracts have been used to treat diarrhea and wounds [46].

Antinutrients, often perceived as detrimental due to their impact on nutrient absorption, play a dual role in plant biology and human health. While they serve as essential chemical defense mechanisms for plants, they also offer potential health benefits, including antioxidant, antimicrobial, and immunomodulatory properties. Understanding their biological functions allows for informed dietary strategies, such as food preparation techniques that reduce negative effects while preserving beneficial properties. Rather than being solely harmful, antinutrients contribute to a complex interplay between vegan and vegetarian diets and human physiology, reinforcing the importance of a balanced and well-managed approach to plant nutrition.

## 4. Effects of Antinutrients on the Nervous System

Antinutrients in plant-based foods are commonly recognized for their effects on digestion and nutrient absorption, but growing evidence suggests they may also have significant implications for neurological health [47]. These compounds, such as oxalates and lectins, have been studied for their potential to directly affect neural tissues or indirectly influence brain function through pathways such as inflammation and the gut–brain axis [48]. While their impact on the nervous system is a relatively new area of research, it holds particular importance for individuals adhering to vegan and vegetarian diets, where reliance on plant-based foods is heightened. In such dietary patterns, understanding how antinutrients interact with neurological processes is critical to mitigating potential risks and ensuring optimal cognitive and nervous system health [49]. By examining the pathways through which antinutrients exert their effects and exploring strategies to counteract these impacts, it is possible to maintain the health-promoting aspects of vegan and vegetarian diets while addressing concerns related to neurological well-being [49].

In line with this, antinutrients can influence neurological health through two distinct mechanisms: direct biochemical interactions with neurological systems and indirect effects mediated by the gut–brain axis [50]. Among direct effects, for individuals with an impaired oxalate metabolism or excessive dietary intake, oxalates may accumulate in the body, leading to systemic complications. Emerging evidence links oxalates to neurotoxicity through mechanisms such as the formation of oxalate deposits in neurological tissues and the promotion of oxidative stress [51]. These processes may disrupt cellular integrity, impair mitochondrial function, and contribute to neural damage. Although rare, elevated oxalate levels have been implicated in neurological conditions such as neuropathy and, in extreme cases, encephalopathy. These effects are typically associated with underlying metabolic disorders, such as primary hyperoxaluria or kidney dysfunction, highlighting the need for careful dietary management in at-risk populations [52]. Moreover, lectins have been proposed as potential neuroinflammatory agents. These proteins have a high affinity for glycoproteins on cell surfaces, allowing them to interact with various tissues, including those of the nervous system [53]. Under certain conditions, lectins may cross the blood–brain barrier or interact directly with neural cells, triggering localized or systemic inflammatory responses. Chronic low-grade inflammation in the brain, often referred to as neuroinflammation, is a well-recognized contributor to neurological disorders such as Alzheimer’s disease, Parkinson’s disease, and other forms of cognitive decline [13]. The ability of lectins to perpetuate inflammatory signaling pathways underscores their potential role in exacerbating these conditions, particularly in individuals with predisposing genetic or environmental factors [54].

Furthermore, the gut–brain axis serves as a critical mediator of the indirect effects of antinutrients on neurological health. This bidirectional communication system links the gastrointestinal tract and the central nervous system, with the gut microbiota playing a pivotal role in maintaining this connection [54]. In line with this, lectins have been shown to compromise gut barrier integrity by increasing intestinal permeability. This disruption allows pro-inflammatory molecules, bacterial endotoxins, and undigested food particles to translocate into the bloodstream, triggering systemic inflammatory responses. These inflammatory mediators can cross the blood–brain barrier, amplifying neurological inflammation [55]. Such cascades are believed to exacerbate or even initiate neuroinflammatory conditions, contributing to a range of disorders, including anxiety, depression, and neurodegenerative diseases. Inflammation originating from gut dysbiosis has also been implicated in altering neurotransmitter synthesis and signaling, further impacting mood and cognitive function. The interplay between direct and indirect pathways highlights the impact of antinutrients on neurological health [56]. While compounds like oxalates and lectins can directly affect neural tissues or inflammation, their effects are often amplified through disruptions to gut health and the gut–brain axis. These findings underscore the importance of dietary strategies to minimize the neurological risks associated with antinutrients while maintaining the benefits of vegan and vegetarian diets [57]. While antinutrients are often regarded as inhibitors of nutrient absorption with potential adverse health effects, emerging evidence suggests they may also confer neuroprotective benefits under specific conditions. These protective roles are largely attributed to their antioxidant and anti-inflammatory properties, which may play a crucial role in safeguarding neural tissues from damage associated with oxidative stress and chronic inflammation. Phytates are a notable example of antinutrients with neuroprotective potential. These compounds act as potent antioxidants by chelating pro-oxidant metals such as iron and copper, which catalyze the formation of reactive oxygen species. By reducing the availability of these metals for ROS generation, phytates help mitigate oxidative stress [58,59]. Additionally, phytates may inhibit lipid peroxidation, a process in which free radicals attack neural cell membranes, further contributing to their neuroprotective role [60]. Similarly, polyphenolic compounds like tannins demonstrate significant neuroprotective properties. Furthermore, their anti-inflammatory effects may counteract chronic low-grade neuroinflammation, which is implicated in cognitive disorders such as mild cognitive impairment and dementia. Also, improving cerebral blood flow and oxygenation is critical for maintaining cognitive function. This dual role of antinutrients remains an area of active research [61]. While their negative effects on nutrient bioavailability are well documented, their capacity to reduce oxidative stress and inflammation poses a new paradigm. Future studies are needed to clarify the conditions under which these compounds exert their neuroprotective effects, as well as their long-term impact on cognitive health. These insights could pave the way for dietary strategies that maximize the benefits of antinutrients while minimizing their drawbacks, particularly in plant-based dietary patterns.

Therefore, addressing the potential neurological effects of antinutrients requires targeted nutritional strategies to reduce their impact while maintaining the benefits of vegan and vegetarian diets [49]. Through employing preparation techniques, ensuring dietary balance, and enhancing gut health, it is possible to mitigate the risks associated with antinutrients and support optimal neurological function. As previously established in this review, a variety of food preparation methods can significantly decrease the concentration of antinutrients in plant-based foods, thereby reducing their potential neurotoxic effects [62]. Further on, ensuring a diverse and balanced diet is essential to minimize the adverse effects of antinutrients on neurological health. Vegan and vegetarian diets should incorporate fortified or supplemented sources of essential nutrients to counteract potential deficiencies exacerbated by antinutrient activity. For example, consuming foods high in vitamin C, such as citrus fruits or bell peppers, enhances the absorption of non-heme iron, counteracting the inhibitory effects of tannins and phytates. Additionally, the inclusion of antioxidant-rich foods, such as berries, dark leafy greens, and polyphenol-containing beverages like green tea, can mitigate oxidative stress associated with antinutrients. These antioxidants play a crucial role in protecting neural tissues from free radical damage and supporting overall brain health [62]. Moreover, maintaining a robust and diverse gut microbiota is essential for mitigating the neurological effects of antinutrients. Incorporating probiotic-rich foods, such as fermented vegetables, yogurt alternatives, or kombucha, and prebiotic fibers found in foods like bananas, garlic, and oats, can support the integrity and resilience of the gut barrier. Addressing gut permeability through these dietary interventions helps reduce the translocation of inflammatory molecules [63,64].

Antinutrients such as oxalates, phytates, tannins, and lectins can negatively affect the nervous system through mechanisms such as nutrient depletion, oxidative stress, and inflammation.

To mitigate these effects, practical dietary recommendations include the following:

- Soaking and Sprouting:
  This reduces phytates and tannins by soaking legumes, grains, and seeds before cooking. Additionally, sprouting enhances the bioavailability of critical nutrients like iron and zinc, which are essential for neurological health.
- Cooking at High Temperatures:
  This deactivates lectins and protease inhibitors in beans and legumes through boiling or steaming. This method ensures safe consumption and reduces gastrointestinal and neurological stressors.
- Incorporating Vitamin C-Rich Foods:
  Pairing iron-rich plant foods with vitamin C sources (e.g., spinach with citrus fruits) enhances iron absorption, counteracting the inhibitory effects of phytates.
- Supplementation:
  Using algae-based DHA/EPA supplements offsets the limited conversion of ALA in plant-based diets, while vitamin B12 supplementation supports myelin synthesis and cognitive function.
- Fortified Foods:
  Consuming fortified plant-based alternatives (e.g., fortified cereals or milks) addresses common deficiencies that are exacerbated by antinutrient interactions.

These strategies reduce the adverse impacts of antinutrients on the nervous system while preserving the nutritional benefits of vegan and vegetarian diets. Further research is essential to refine these approaches and explore their long-term neurological implications.

## 5. Micronutrient Deficiencies of Vegan and Vegetarian Diets in Neurological Health

The adoption of vegan and vegetarian diets has transformed dietary patterns worldwide, with significant demographic and cultural influences shaping the prevalence and nutritional outcomes of these lifestyles. In the United States, an estimated 10 million adults identify as vegetarian, with approximately 3.3 million adhering to a vegan diet, representing 1% of the total population. Across Europe, the prevalence of vegetarianism and veganism has doubled over the last decade, with Germany reporting 10% adherence and the United Kingdom seeing similar trends [65]. These shifts reflect growing awareness of ethical, environmental, and health considerations. However, the implications for neurological health, particularly concerning micronutrient deficiencies, remain a significant public health challenge.

Micronutrient inadequacies in vegan and vegetarian populations have profound implications for brain health. Vitamin B12 deficiency, one of the most pervasive issues, affects 6% of the general population in the United States, but the prevalence is significantly higher in vegans, with up to 52% showing subclinical or clinical signs of deficiency [19]. One key study by Krajcovicova-Kudlackova et al. investigated vitamin B12 deficiency among Czech vegans who did not use B12 supplements. The study found a high prevalence of deficiency in this population, emphasizing the crucial role of supplementation. The results align with broader research indicating that B12 deficiency is widespread among vegans, with prevalence rates ranging from 11% to 90% depending on age and dietary habits. Furthermore, a systematic review of 18 studies conducted across Europe, America, Africa, and Asia reported deficiency rates of 62% in pregnant women, 25–86% in children, 21–41% in adolescents, and 11–90% in older adults. These findings highlight the necessity of B12 supplementation in vegan and vegetarian diets to prevent neurological and hematological complications [66,67].

Elevated homocysteine levels associated with B12 deficiency have been linked to a 50–70% increased risk of Alzheimer’s disease and vascular dementia, underscoring the critical role of this nutrient in neurological protection. In infants and toddlers raised on vegan diets, B12 deficiency correlates with developmental delays, reduced brain volume, and impaired motor function, emphasizing the importance of maternal supplementation during pregnancy and lactation [68]. Thus, the relationship between vitamin B12 levels and cognitive health has been widely studied, particularly in the context of Alzheimer’s disease. Research suggests that vitamin B12 plays a crucial role in neurological function, and its deficiency has been associated with cognitive decline and neurodegenerative diseases. A recent meta-analysis reviewed five randomized controlled trials investigating the effects of vitamin B12 and folic acid supplementation in patients with Alzheimer’s disease. The study concluded that supplementation may reduce homocysteine levels, which are linked to a slower progression of the disease. However, the cognitive and functional benefits of supplementation remain inconsistent, highlighting the need for further research to establish definitive clinical recommendations [69].

Another study explored the association between vitamin B12 deficiency and neurocognitive disorders, finding that individuals with lower B12 levels exhibited higher levels of homocysteine, a risk factor for neurodegeneration. The findings suggest that adequate vitamin B12 intake may be crucial for preserving cognitive function and potentially reducing the risk of Alzheimer’s disease [70].

Furthermore, research published in 2018 indicates that vitamin B12 is essential for maintaining myelin integrity and neurotransmitter function [71]. The study highlights that chronic B12 deficiency may contribute to structural and functional brain alterations observed in Alzheimer’s disease. The authors emphasize the need for early detection and supplementation strategies, particularly among older adults and individuals following vegan and vegetarian diets. Overall, these findings reinforce the importance of maintaining sufficient vitamin B12 levels to support cognitive health. While evidence suggests a potential protective role of B12 against Alzheimer’s disease, further well-designed longitudinal studies are needed to clarify the mechanisms and efficacy of supplementation in preventing cognitive decline [71].

The impact of iron deficiency, particularly non-heme iron from plant sources, further complicates the neurological risks associated with vegan and vegetarian diets. Iron deficiency anemia affects an estimated 1.6 billion people globally, with prevalence rates as high as 30% among vegans in Western countries and up to 60% in regions like South Asia [6,67]. Iron is essential for oxygen transport and neurotransmitter synthesis, and its deficiency has been associated with reduced cognitive performance, fatigue, and developmental delays in children. Longitudinal studies indicate that children with early-life iron deficiency are more likely to experience long-term deficits in IQ and attention span, demonstrating the far-reaching consequences of inadequate iron intake [68].

Zinc inadequacy is another critical issue, affecting approximately 35% of vegans globally [6]. Zinc plays a pivotal role in synaptic plasticity, neurogenesis, and immune function, and its deficiency has been linked to impaired learning, memory, and increased susceptibility to infections. In regions where phytate-rich foods dominate the diet, zinc bioavailability is further reduced, exacerbating the risk of deficiency. Research in populations with high plant-based food consumption highlighted a 15–30% higher incidence of suboptimal zinc levels compared to those consuming omnivorous diets, with corresponding increases in cognitive and immune-related disorders [6,68].

Omega-3 fatty acid deficiencies, particularly of DHA and EPA, are another pressing concern in vegan and vegetarian populations. ALA, the plant-based precursor to DHA and EPA, has a conversion rate of less than 10%, with even lower efficiency observed in older adults and those with metabolic disorders. Studies have shown that DHA levels in vegans are approximately 50% lower than those in omnivores, correlating with a higher prevalence of depression, anxiety, and cognitive decline [69]. DHA is critical for neuronal membrane integrity and the resolution of neuroinflammation, both of which are vital for preventing neurodegenerative diseases.

Iodine deficiency, particularly in European countries where iodized salt usage is declining, presents additional neurological risks. Up to 80% of vegans in Europe exhibit insufficient iodine levels, leading to hypothyroidism, cognitive slowing, and depression. Thyroid hormones, dependent on iodine, are essential for brain development and function, and even mild deficiencies during pregnancy can result in lower IQ scores in offspring. Seaweed consumption and fortified products can mitigate these risks, but inconsistent availability and cultural barriers to consumption limit their efficacy in certain regions [72,73].

Compounding these deficiencies are socioeconomic and demographic disparities that exacerbate the risks for certain populations. In low-resource settings, where vegan and vegetarian diets are often adopted out of necessity rather than choice, limited access to fortified foods and supplements further heightens the risk of malnutrition [74]. For example, in South Asia, where vegetarianism is culturally entrenched, combined deficiencies in iron, zinc, and vitamin B12 affect up to 70% of the population, contributing to high rates of stunting, cognitive impairment, and maternal mortality [75]. These disparities underscore the need for tailored public health strategies to address nutrient deficiencies in vegan and vegetarian diets.

The implications of these deficiencies extend beyond individual health, affecting public health systems and economic productivity. The global economic burden of iron deficiency alone is estimated at USD 70 billion annually due to lost productivity and healthcare costs [72]. Similarly, untreated vitamin B12 deficiency contributes to increased healthcare utilization for neurological and psychiatric conditions, particularly among aging populations. Addressing these deficiencies through targeted interventions, such as fortification, supplementation, and the biofortification of crops, is essential for mitigating these costs and improving population health outcomes [73]. Emerging technologies and public health initiatives offer promising solutions to these challenges. The fortification of plant-based foods with B12, iron, zinc, and iodine has proven effective in reducing deficiency rates, particularly in high-risk populations [76]. Algal-based DHA and EPA supplements provide a sustainable and ethical alternative to fish oil, addressing the omega-3 needs of vegans without compromising environmental sustainability. Biofortified crops, such as zinc-enriched rice and low-phytate wheat, represent innovative approaches to improving micronutrient bioavailability while preserving the environmental benefits of plant-based agriculture [48]. Thus, educational campaigns emphasizing the importance of dietary planning and supplementation are equally critical. Many individuals adopting vegan or vegetarian diets lack awareness of the potential for nutrient deficiencies and the steps necessary to prevent them. Public health initiatives must prioritize education, particularly for vulnerable groups such as pregnant women, children, and the elderly, who face the greatest risks from micronutrient inadequacies.

Despite the challenges, vegan and vegetarian diets have the potential to support neurological health when appropriately managed. Their high levels of antioxidants, polyphenols, and fiber confer protective effects against oxidative stress and chronic inflammation, both of which are implicated in neurodegenerative diseases. By addressing the gaps through supplementation, fortification, and dietary planning, vegan and vegetarian diets can be optimized to promote both individual and public health. Ongoing research and interdisciplinary collaboration will be essential to advancing our understanding of these diets and their implications for neurological well-being.

**Figure 1:**
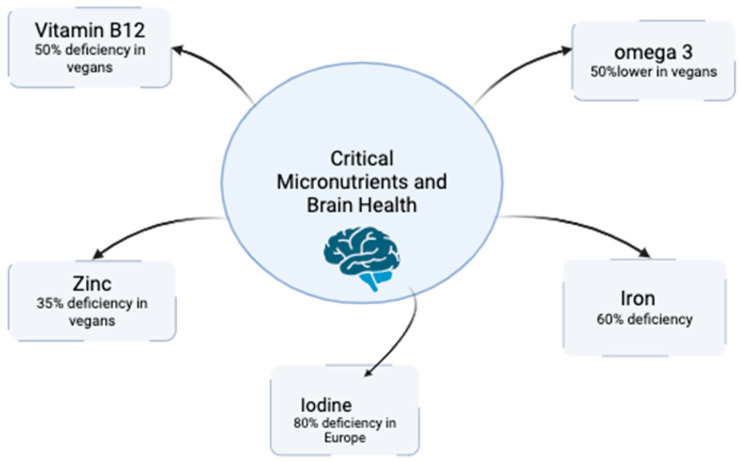
Critical micronutrient deficiencies in vegan and vegetarian diets.

The challenges of nutrient deficiencies associated with vegan and vegetarian diets have spurred innovations such as biofortification and personalized nutrition, which provide promising solutions to optimize nutrient intake and neurological health. Biofortification, an agricultural innovation, enhances the nutritional quality of crops through genetic modification or agronomic practices. Examples include zinc-enriched rice and low-phytate wheat, which are designed to improve the bioavailability of essential nutrients like iron and zinc. These crops are particularly beneficial in regions where access to fortified foods or supplements is limited, offering a sustainable and accessible solution to reduce micronutrient deficiencies and their associated cognitive and neurological impacts (**Figure 1**).

## 6. Plant Phytonutrients and Neurological Health

The relationship between diet and neurological health has gained significant attention in recent years. Among the various dietary patterns, vegan and vegetarian diets are diets that have been gaining in popularity. These diets are characterized by the exclusion of animal products and a reliance on plant-based foods such as fruits, vegetables, legumes, grains, nuts, and seeds. Thus, they are particularly rich in phytonutrients, which are bioactive compounds found in plants. These diets are abundant in polyphenols, flavonoids, vitamins, and other essential nutrients that have been shown to have potential neuroprotective effects [77]. These effects are mediated through several mechanisms, including antioxidant activity, anti-inflammatory properties, and the modulation of signaling pathways.

Phytonutrients, including flavonoids and polyphenols, play a crucial role in supporting neurological health through several biochemical mechanisms. These mechanisms involve the modulation of oxidative stress, inflammation, and the gut–brain axis, which collectively contribute to neuroprotection.

1. Oxidative Stress Reduction: Flavonoids and polyphenols act as potent antioxidants, neutralizing reactive oxygen species (ROS) and reducing lipid peroxidation in neuronal membranes. This action is critical for maintaining cellular integrity and preventing damage associated with neurodegenerative diseases like Alzheimer’s and Parkinson’s.
2. Inflammatory Pathway Modulation: These phytonutrients activate the Nrf2/ARE signaling pathway, which upregulates the expression of antioxidant enzymes such as superoxide dismutase and catalase. Additionally, they inhibit pro-inflammatory pathways mediated by NF-κB, reducing chronic inflammation that is often implicated in cognitive decline.
3. Gut–Brain Axis Interaction: Polyphenols, such as curcumin and epigallocatechin gallate (EGCG), enhance gut microbiota diversity by promoting beneficial bacteria. This improvement in gut health indirectly supports neurological function by reducing systemic inflammation and improving neurotransmitter synthesis, including serotonin and dopamine.
4. Direct Neuroprotective Effects: Certain flavonoids, like quercetin and resveratrol, exhibit direct neuroprotective effects by crossing the blood–brain barrier and protecting neurons from apoptosis. Their roles in enhancing synaptic plasticity and memory formation have been substantiated in recent research.

Oxidative stress is a major contributor to neurodegenerative diseases such as Alzheimer’s and Parkinson’s disease. Hence, oxidative stress linked to mitochondrial dysfunction and neuroinflammation is a hallmark of neurodegenerative diseases, primarily due to the CNS’s unique metabolic features. The brain consumes large amounts of oxygen even at rest, leading to increased production of reactive oxygen and nitrogen species (ROS and RNS) through specific neurochemical reactions. Additionally, aging contributes to the accumulation of metabolites like pathogen-associated molecular patterns (PAMPs) and damage-associated molecular patterns (DAMPs) [78]. These processes interact causally, creating a cycle of persistent oxidative stress. This cycle triggers neuronal apoptosis and glial cell neurotoxicity, ultimately leading to neuronal dysfunction, damage, or progressive loss [79]. For example, the development of Parkinson’s disease likely arises from a combination of intestinal dysbiosis, inflammation, oxidative stress, mitochondrial dysfunction, alpha-synuclein clumping, and the degeneration of dopaminergic neurons in the nigrostriatal pathway [80].

Polyphenols are a diverse group of phytochemicals found in high concentrations in fruits, vegetables, tea, coffee, and wine [81]. They are known for their antioxidant properties, which help in reducing oxidative stress, a key factor in the development of neurodegenerative diseases [82]. Therefore, phytonutrients like polyphenols and vitamins C and E help in neutralizing free radicals, thereby reducing oxidative stress to neurons [83], as they act as reactive oxygen species scavengers. Studies have shown that polyphenols can modulate brain function by influencing signaling pathways, reducing inflammation [84], and protecting neurons from damage due to their inhibition of signal transduction mechanisms of pro-inflammatory intermediaries and cellular inflammatory pathways at the molecular level. Thus, as antioxidants, they reduce lipid, protein, carbohydrate, and DNA oxidation [85], as well as increase glutathione peroxidase and superoxide dismutase activity. For instance, resveratrol, a polyphenol found in grapes and berries, activates the SIRT1 pathway, which is involved in cellular stress resistance and longevity, known as antiaging proteins [86]. Additionally, this has been highlighted by their neuroprotective properties in Alzheimer’s disease models [87]. It neutralizes O2−•, OH−•, and lipid hydroperoxyl radicals. Similarly, epigallocatechin gallate (EGCG) defends against the neurotoxin MPTP, which induces Parkinson-like symptoms, by inhibiting its absorption or neutralizing radicals generated by MPTP [88].

Moreover, several phytonutrients can influence various signaling pathways involved in brain function. For example, resveratrol, curcumin, and a flavonoid, quercetin, have been shown to activate the Nrf2 (nuclear factor E2-related factor 2) pathway, which enhances the expression of antioxidant enzymes and protects against neuronal damage [89]. Nrf2 is a key regulator of antioxidant enzymes that protect neurons from oxidative stress and inflammation. The Nrf2/antioxidant response element (ARE) pathway has been identified as a potential target for diseases caused by oxidative stress. Dietary polyphenols may influence Nrf2 signaling by affecting different kinases upstream of Nrf2. These polyphenols also activate Nrf2 expression and its downstream targets, including heme oxygenase, superoxide dismutase, and catalase, which help combat oxidative stress and, consequently, regulate inflammation [90].

Additionally, polyphenols have not only been noted for their potential to reduce oxidative stress, but also to improve the brain microbiota axis. For example, the polyphenol curcumin, found in turmeric, enhances the activity of antioxidant enzymes such as superoxide dismutase and catalase [91], while it also plays a significant role in promoting gut function and overall metabolism through multiple mechanisms. It reduces the expression of the chaperone protein BiP, which helps modulate endoplasmic reticulum stress and the inflammatory response in human intestinal epithelial cell lines [92]. By alleviating endoplasmic reticulum stress and inflammation, curcumin supports the integrity and function of the intestinal barrier. Additionally, curcumin enhances the signaling of critical neurotransmitters in the hippocampus and peripheral intestinal system. These include brain-derived neurotrophic factor (BDNF), serotonin, and cAMP response element-binding protein (CREB), which are essential for maintaining neuronal health, mood regulation, and gut–brain communication [93]. Through these combined effects, curcumin not only improves gut health, but also strengthens the gut–brain axis, highlighting its potential as a therapeutic compound for both intestinal and neurological disorders. Moreover, curcumin offers neuroprotection in neurodegenerative diseases by preventing α-synuclein aggregation in Parkinson’s disease, reducing ROS-induced Cox-2 in amyotrophic lateral sclerosis, alleviating multiple sclerosis symptoms, minimizing brain injuries, and suppressing inflammatory mediator overexpression during neuroinflammation [92]. Similar results were found in epigallocatechin-3-gallate (EGCG), a major bioactive compound present in green tea, which promotes the growth of beneficial gut bacteria and reduces gut inflammation, which may have neuroprotective effects [94].

Flavonoids, a subgroup of polyphenols, are found in a variety of plant-based foods, including berries, apples, onions, and dark chocolate. They have been shown to improve cognitive function and memory by enhancing synaptic plasticity and reducing neuroinflammation [95]. Flavonoids such as quercetin and apigenin have demonstrated potential in reducing amyloid-beta accumulation, a hallmark of Alzheimer’s disease [96]. Quercetin, found in apples and onions, has been shown, in the recent literature, after having been developed in an animal model, to be capable of inhibiting the aggregation of amyloid-beta peptides and reducing oxidative stress by modulating the Nrf2 and phosphatidylinositol 3-kinase/Akt pathway [97]. Moreover, quercetin has been shown, in the recent literature, to inhibit signaling pathways associated with inflammation promotion, such as nuclear factor-kappa B (NF-κB), as well as to modulate various cells’ survival and cell cycle genes [98].

Vegan and vegetarian diets, rich in phytonutrients such as polyphenols, flavonoids, and vitamins, offer significant potential for enhancing neurological health. These diets provide a variety of bioactive compounds that can reduce oxidative stress, inflammation, and modulate signaling pathways, thereby protecting against neurodegenerative diseases. However, it is essential to ensure adequate intake of certain nutrients, such as vitamin B12, through supplementation. Further research is needed in order to completely understand the mechanisms through which these phytonutrients produce their neuroprotective effects and to optimize dietary recommendations for neurological health.

## 7. Inflammation and Oxidative Stress in Vegan and Vegetarian Diets

Vegan and vegetarian diets come in several forms, each with distinct features. Vegan diets are strictly plant-based, excluding all animal products. Lacto-ovo vegetarian diets include plant-based foods along with dairy products and/or eggs. Pesco-vegetarian or pescatarian diets are mainly plant-based but also incorporate fish and seafood, with the option of including eggs and dairy. All these vegetarian diets exclude meat, such as beef, pork, lamb, chicken, and other poultry, as well as related meat products [99]. Vegetarian and vegan diets are associated with anti-inflammatory effects partly because they exclude meat products, which contain pro-oxidant substances. Meat, especially processed meat, can lead to oxidative stress due to the presence of heme iron and other compounds, which promote lipid peroxidation, enhancing the proinflammatory environment [100].

Recently, vegetarian and vegan diets have gained popularity not only for ethical and environmental reasons, but also for their potential health benefits [74]. Among these benefits, their anti-inflammatory and antioxidant properties are particularly remarkable. Hence, vegetarian and vegan diets have been pointed out by the previous literature as rich in various bioactive compounds that contribute to their health benefits.

Firstly, polyphenols have been described as an important key factor in reducing oxidative stress by neutralizing free radicals. These compounds are abundant in fruits, such as apples, blueberries, strawberries, and grapes, and in legumes, nuts, olive oil, tea, coffee, chocolate, and wine. Moreover, flavonoids, a subgroup of polyphenols, also have been associated with anti-inflammatory effects by inhibiting the production of pro-inflammatory cytokines. These bioactive products are classified by their chemical structure, resulting in different types of compounds [101]. Flavanols, such as quercetin, kaempferol, and myricetin, are found in vegetables such as tomatoes, onions, and broccoli, as well as in fruits including grapes and apples. Flavones like luteolin and apigenin are present in spices such as parsley, celery, and thyme, as well as in vegetables such as hot peppers. Flavanones such as naringenin and hesperetin are abundant in several fruits including grapefruits, oranges, lemons, citrus peels, and citrus fruits [102]. Anthocyanins, including cyanidin, malvidin, petunidin, and peonidin, are found in red fruits, such as strawberries, raspberries, blueberries, bilberries, elderberries, black currants, cherries, pomegranates, red wine, and red onions. Isoflavones such as genistein may be found in soy and tofu. Finally, flavanols may be present in green tea, red grapes, and red wine. In this line, polyphenols have been shown by their activity to inhibit the activation of nuclear factor kappa B (NF-κB), a protein complex that plays a key role in regulating the immune response to infection and inflammatory events [103]. By inhibiting NF-κB, polyphenols and flavonoids reduce the expression of inflammatory genes, including COX-2 and VEGF (vascular endothelial growth factor), as well as several pro-inflammatory substances, including cytokines, such as IL-1, IL-2, IL-6, and TNF- α, and chemokines, such as IL-8, MIP-1α, and monocyte-chemotactic protein-1 (MCP-1) [104].

Vegetarian and vegan diets are typically high in vitamins C and E, which are potent antioxidants, as they are key radical-scavenging antioxidants in vivo [105]. Thus, vitamin E serves as the primary lipophilic antioxidant, while vitamin C functions as the essential hydrophilic radical scavenger [106]. Hence, their activity reduces free radicals, decreasing the inflammatory environment. This fact may also be explained due to their capability of inhibiting the production of various proinflammatory cytokines, including tumor necrosis factor-alpha (TNF-α), interleukin-1 (IL-1), IL-6, and (MCP-1) [65]. Moreover, both vitamins have been recently described as potential reducers of cyclooxygenase 2 activity. Furthermore, vitamin E has been shown to be capable of protecting cell membranes from oxidative stress, as it offers robust protection against lipid peroxidation, DNA mutations, and mitochondrial damage [66]. In line with this, nutritional epidemiological studies have demonstrated that the dietary intake of vitamin E, or the combined use of α-tocopherol and γ-tocopherol, is linked to a delay in age-related cognitive decline and a reduced risk of Alzheimer’s disease. Vitamin C may be found in several fruits, including citrus fruits and strawberries, as well as in vegetables such as peppers and tomatoes [67]. On the other hand, vitamin E is present in nuts, seeds, avocado, mango, kiwi fruit, and green leafy vegetables [107].

These diets, due to their high fiber concentrations, have also been highlighted by the recent literature as anti-inflammatory diets. High fiber intake from whole grains, fruits, and vegetables aids in the production of short-chain fatty acids (SCFAs) during fermentation in the gut, which have anti-inflammatory effects [68]. This activity is related to acetate, propionate, and butyrate production from different gut microbiota. More specifically, butyrate has been related to the inhibition of the production of inflammatory cytokines, including TNF-α, MCP-1, and IL-6 [69]. Additionally, propionate administration has reduced inflammatory mediator levels, including IL-4, IL-5, and IL-17A, in animal models [70].

Phytosterols, present in nuts, seeds, and legumes, can help reduce cholesterol levels by inhibiting its absorption in the small intestine, which may have anti-inflammatory effects. This fact may be explained due to their ability to reduce the reabsorption of cholesterol and bile acids in the small intestine, leading to an increased uptake of LDL cholesterol by the liver, reducing LDL cholesterol plasma levels [71]. This mechanism is also shared with fiber, which is also related to a reduction in LDL cholesterol levels due to its capability to reduce the reabsorption of cholesterol [108]. These compounds have been shown to reduce oxidative stress by enhancing the activity of antioxidant enzymes. This property has been linked to stigmasterol, an unsaturated phytosterol, which has recently been related to a reduction in the release of tumor TNF-α, nitric oxide (NO), IL-1β, and IL-6, while also inhibiting cyclooxygenase-2 (COX-2), contributing to a decrease in inflammation [109]. Moreover, recent studies have highlighted how stigmasterol reduces the levels of ROS, as it enhances the activities of antioxidant enzymes as catalase, superoxide dismutase, and nitric oxide synthase. Additionally, it reduces ROS production [110].

Carotenoids, pigments found in colorful fruits and vegetables like carrots, sweet potatoes, and spinach, have also been associated with antioxidant properties. Carotenoids such as beta-carotene, lutein, and zeaxanthin help protect cells from oxidative stress and support immune function [111]. These compounds can reduce inflammation by inhibiting the production of pro-inflammatory cytokines and enhancing the immune response. Beta-carotene, for example, has been shown to reduce levels of C-reactive protein (CRP) and IL-6. These compounds can neutralize free radicals and protect cells from oxidative stress. Lutein and zeaxanthin, for example, are known to protect the eyes from oxidative stress and reduce the risk of age-related macular degeneration, as they act by increasing the ability to scavenge retinal ROS, with zeaxanthin being more effectively scavenged than lutein [112].

Another bioactive compound, sulforaphane, has been shown to reduce markers of inflammation, as it activates the Nrf2 (nuclear factor erythroid 2-related factor 2) pathway, which enhances the expression of antioxidant enzymes and reduces oxidative stress and inflammation [113]. Nevertheless, this compound is not directly found in plants, including cruciferous vegetables. Instead, plants of the Brassica genus contain glucoraphanin, a biologically inactive precursor stored in cell vacuoles. An enzyme called myrosinase, stored in a separate compartment, activates sulforaphane production. When plant cells are damaged, such as through chewing or cutting, glucoraphanin and myrosinase mix, triggering an enzymatic reaction that produces sulforaphane [113]. The Nrf2 pathway, though not fully understood, involves these key components. Nrf2 is sequestered in the cytoplasm by Keap-1, a cysteine-rich protein acting as a redox sensor. Under oxidative or electrophilic stress, cysteine residues in Keap-1 detect changes, disrupting its ability to bind Nrf2. Freed Nrf2 translocates to the nucleus, where it binds Antioxidant Response Elements (AREs) in gene promoters, often dimerizing with small Maf proteins to form a transactivation complex. This upregulates genes critical for cellular defense, including those boosting γ-glutamyl-cysteine synthetase, the enzyme regulating glutathione (GSH) synthesis. Elevated GSH levels enhance antioxidant capacity, protecting cells from oxidative stress [114,115].

Lastly, vegetarian and vegan diets have also been associated with maintaining cellular health and reducing inflammation, as these diets often provide significant amounts of several minerals, including magnesium and potassium. Magnesium, found in nuts, seeds, and whole grains, has been shown to reduce markers of inflammation such as CRP [116].

**Figure 2:**
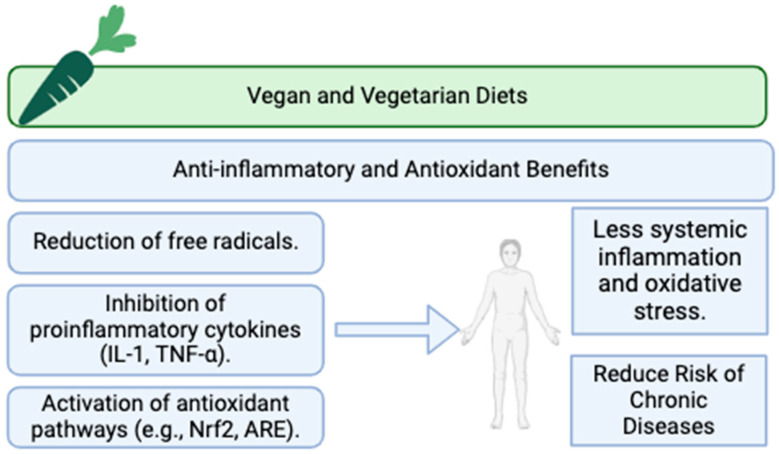
Anti-inflammatory and antioxidant effects of vegan and vegetarian diets.

In summary, vegetarian and vegan diets offer significant anti-inflammatory and antioxidant benefits due to their high content of polyphenols, flavonoids, vitamins, fiber, carotenoids, phytosterols, isoflavones, and sulforaphane. These components work synergistically to reduce inflammation and oxidative stress, contributing to overall health and potentially lowering the risk of chronic diseases. Further research is needed to fully understand the mechanisms and long-term effects of these diets, but current evidence supports their role in promoting health and well-being (**Figure 2**).

## 8. Vegetarian Diets on Mood Regulation and Mental Well-Being

Vegetarianism has attained increased prominence in recent years. Although the health benefits of a vegetarian diet are well documented [117], its impact on the quality of life of adherents need further investigation. Quality of life pertains to an individual’s subjective assessment of well-being and functionality, encompassing four primary domains: physical, psychological, social, and environmental. The adoption of a vegetarian diet, while a dietary pattern, may affect and be affected by all of these categories, either positively or negatively [118]. The psychological domain refers to affective states, self-worth, body image, and cognitive processes such as learning, memory, and focus. Various facets of vegetarianism may either affect or be affected by psychological considerations [119]. Considering this, in psychological research, vegans and vegetarians are frequently lumped together and typically compared to omnivores, despite the fact that their diets and lifestyles differ greatly from one another [120]. Thus, generally, with a controlled and balanced vegan or vegetarian diet, it is entirely possible to maintain a healthy lifestyle and even gain many physical and mental benefits. Maybe the key lies in proper planning to meet all the body’s nutritional needs and prevent potential deficiencies [121].

### 8.1. Positive Influences

Steering clear of meat and other animal products can increase the positive emotions that result from a person adopting a mindset that supports their beliefs [122]. The beneficial psychological effects extend beyond the individual level since they can strengthen social ties with people who share similar beliefs and actions [123]. Being a vegetarian may be more than just choosing a dietary pattern, as Rosenfeld and collaborators pointed out; it also creates a new social identity that affects how people think, act, and interact with others. Changing to a plant-based diet can improve happiness and well-being, which may enhance a person’s quality of life [124]. A study carried out by trackinghappiness.com (1 February 2025) indicates that vegans are happier than the general population. Compared to meat eaters, vegans report 7% higher levels of happiness. It is interesting to note that happier people think they are more likely to become vegan in the future [125]. In line with this, analyzing a sample of university students in Spain, it has also been discovered that some happiness constructs (tranquility, fulfilment, and virtue) are positively related to vegetarianism, while others are inversely related (enjoyment and stoicism) [126]. Bertella reported, in 2020, that, in small groups, vegans experience sensual gratification, enjoyment, conviviality, and meaningfulness, but they also feel alone and frustrated [127]. Also, a recent scoping review focused on examining the connection between eating or not eating meat and psychological well-being, and it was reported that, according to most studies, there are no differences in positive psychological variables between meat eaters and meat abstainers [128]. Nevertheless, a study with 39 omnivores compared dietary patterns (omnivorous, pescatarian, vegetarian) over two weeks. While mood scores remained stable for omnivores and pescatarians, vegetarians showed significant mood improvements [129]. In another study, Beezhold and collaborators also reported that, despite consuming fewer long-chain omega-3 fatty acids, the vegetarian diet profile did not seem to have a negative impact on mood [130]. Also, a study of 620 adults found that vegans reported lower anxiety (in males) and stress (in females) levels compared to omnivores, with mood benefits linked to vegan and vegetarian diets and healthier eating habits [131].

However, happiness perceptions are typically linked to better health when eating a vegetarian diet [132]. Regarding this, it is known that a well-balanced vegan and vegetarian diet supports optimal health and reduces the risk of chronic diseases [133]. Despite the challenging path, patients reported partial and complete relief from some disease symptoms when they adopted a vegetarian diet [132]. They lived a healthy lifestyle and felt young and energetic. In a similar vein, the strategy carried out by Khaledi-Paveh’s study enhanced people’s mental health and mood [132]. In this regard, veganism is associated with reduced risks of metabolic syndrome and cardiovascular disease, but may pose nutrient deficiency risks [133]. Also, there is a link between vegans and healthier lifestyles, including smoking less, performing more physical activity, and having a higher socioeconomic status, which could also contribute to better mood outcomes [134]. Concretely, a 12-week study of 500 adults with chronic depression and anxiety found that a plant-based diet, exercise, and mindfulness led to significant improvements in mental health, with most participants reporting substantial benefits [135]. Raman et al. also reported that, when combining an advanced meditation program with a vegan diet, a sustained increase in beneficial gut bacteria was found, suggesting potential links to improved mood and psychological processes, warranting further research [136].

In summary, combining a vegan or vegetarian diet with regular exercise can positively impact mood and mental health [137]. Vegan and vegetarian diets, rich in antioxidants and anti-inflammatory compounds, may reduce inflammation linked to psychological disorders [137,138]. Exercise boosts endorphins, further enhancing emotional well-being. Together, they can improve energy levels, reduce stress, and promote better sleep, all of which support mental health. Additionally, the combination fosters a stronger mind–body connection, encouraging healthier habits. However, it is crucial to address potential nutrient deficiencies (e.g., B12, iron, omega-3s) to avoid negative effects on mental well-being [139]. Overall, this pairing can lead to improved mood and overall psychological resilience when nutrition is well balanced.

### 8.2. Negative Influences

Recent research highlights the important role of diet in mental health [140]. Vegetarian diets, in particular, have been associated with various health benefits. However, the impact of plant-based dietary patterns can differ based on how they are defined—ranging from excluding all animal products to emphasizing a high intake of plant-based foods—leading to mixed results [141,142,143]. For example, a recent meta-analysis found that vegetarian and semi-vegetarian diets might elevate the risk of depression, whereas lacto-ovo vegetarian diets showed no significant association with depression [144].

Thus, the psychological effects of vegetarianism are mixed, with some studies linking it to improved mental health while others suggest potential risks. For instance, vegetarian Seventh-day Adventists reported better mood, and South Asian vegetarians in the U.S. had a 43% lower likelihood of depression [145]. Conversely, studies in the U.K. found higher depressive symptoms in men, even after accounting for nutritional and sociodemographic factors [146]. Similarly, research in Turkey observed increased anxiety and eating disorders among adolescents, suggesting that vegetarianism might sometimes be adopted to restrict food intake, potentially linked to pre-existing eating disorders [147]. A recent systematic review found conflicting evidence on the link between vegetarian or vegan diets and depression: 44% of outcomes showed higher depression rates, while 28% indicated mood benefits [148]. However, Walsh’s study highlights that diet quality, whether in meat-based or vegan and vegetarian diets, may influence depressive symptoms. High-quality vegan and vegetarian diets showed a stronger protective effect against depression, warranting further research on this bidirectional relationship [149].

**Figure 3:**
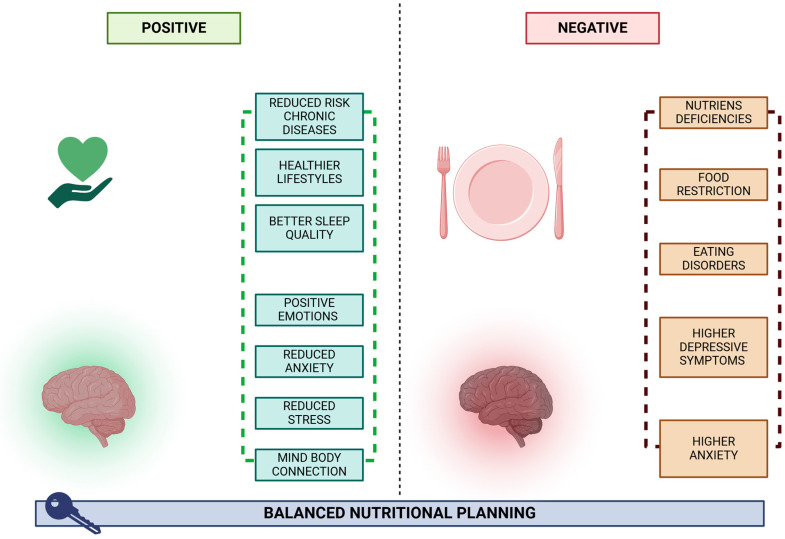
Influence of vegan and vegetarian diets on mood.

More evidence remains inconclusive. Some studies suggest that vegetarians and vegans experience higher depressive symptoms compared to omnivores, with meat abstainers showing more significant symptoms than meat eaters [150]. However, other research indicates that vegan and vegetarian diets may be associated with lower depressive symptoms than omnivorous diets [151]. Systematic reviews also reflect inconsistent findings, suggesting that depression may not be directly linked to vegan and vegetarian diets themselves but, rather, to their quality, as mentioned before [108]. Concretely, factors like food restriction and exclusion of food groups, beyond the absence of meat, may also confound this relationship [152]. An Australian study involving 219 vegans and vegetarians aged 18 to 44 found that high-quality vegan and vegetarian diets correlated with fewer depressive symptoms, while low-quality diets were associated with greater symptoms [153]. These findings align with general observations about diet quality and health, but further research comparing diet quality across plant-based and omnivorous diets is necessary to better understand this relationship (**Figure 3**).

## 9. Influence of Vegan and Vegetarian Diets on Neurodegenerative Diseases

In recent years, vegan diets have gained worldwide popularity, with surveys from 2021 indicating that vegans make up 2–3% of the population in European countries. A global survey of sustainable diet strategies across 150 countries found vegan diets to be the most effective in terms of mortality, health, and environmental outcomes compared to vegetarian, pescatarian, and flexitarian diets. People choose vegan diets for various reasons, including ideological, religious, or medical motivations. Notably, there is evidence suggesting that vegan diets may help prevent neurodegenerative disorders [154]. Neurodegenerative disorders are increasing globally, with dementia cases expected to triple by 2050 [155]. Alzheimer’s disease (AD), the leading cause of dementia, is influenced by non-modifiable factors like age and genetics, and modifiable ones such as diet, physical inactivity, and depression [156]. While there is no cure, addressing modifiable risk factors, particularly diet, can lower AD risk.

Dietary patterns such as Mediterranean, MIND (Mediterranean-DASH Intervention for Neurodegenerative Delay), and DASH (Dietary Approaches to Stop Hypertension) have been shown to enhance brain health. Following the Mediterranean, DASH, or MIND diet may reduce cognitive decline [157,158]. These diets share the commonality of restricting sugar and saturated fat consumption while advocating for a high intake of fruits, vegetables, whole grains, and nuts, alongside a low consumption of red or processed meat. Numerous medical organizations have advocated for a plant-based diet to enhance cognitive health and maybe avert dementia. Adopting a plant-based diet can be a low-risk and advantageous lifestyle modification to preserve cognitive health and mitigate cognitive aging [159].

Nonetheless, plant-based and vegan diets are not equivalent. A vegan diet is mostly plant-based; however, not all vegan and vegetarian diets are classified as vegan. A plant-based diet mostly consists of vegetables, with minimal inclusion of animal products. A whole-food plant-based diet focuses on whole unrefined plant foods while minimizing highly refined items, including bleached wheat, refined sugar, oil, and processed packaged meals. A vegan diet completely excludes all animal products and is a more stringent form of vegetarianism. In addition to abstaining from meat, vegans exclude all products created from or derived from animals, such as dairy, eggs, and honey. Moreover, vegans abstain from utilizing animal products in other aspects of their lives, such as cosmetics, footwear, and apparel [160]. Knowing this, the impact of a plant-based diet on neurological health and cognitive function is widely established [161,162].

### 9.1. Positive Effects

Vegan diets offer a nutrient-rich profile, being abundant in fiber, polyunsaturated fatty acids, and essential vitamins (A, B1, B6, C, E), as well as folate, magnesium, iron, and copper [163]. These nutrients are associated with potential benefits for neurodegenerative disease prevention and management [164].

A cornerstone of vegan diets, fruits and vegetables, as pointed out by Loef and Walach (2022), significantly reduce dementia risk and cognitive decline [155]. Their high antioxidant and anti-inflammatory properties, along with phytochemicals and fiber content, help counteract the pathological processes involved in, for instance, AD [155] and Parkinson’s disease [165,166]. Additionally, this type of diet may reduce inflammation by lowering markers such as C-reactive protein (CRP), particularly with long-term adherence [155]. In this regard, vegan and vegetarian diets also address modifiable neurodegenerative risk factors [166], such as obesity, hypertension, diabetes, and high cholesterol, by promoting a healthy body weight and improving cardiovascular health [167]. Their higher fiber and polyphenol content enhances insulin sensitivity, glycemic control, and metabolic health, which are crucial for cognitive protection [168,169].

Regarding gut microbiota, emerging research highlights the role of GUT in these disease pathogeneses [170]. Vegan and vegetarian diets foster a more diverse and stable gut microbiome, improving the production of beneficial metabolites like short-chain fatty acids (SCFAs). These metabolites support brain health by reducing inflammation and amyloid-beta (Aβ) pathology. Furthermore, Liu et al. remarked that vegan diets lower trimethylamine N-oxide (TMAO) levels [168]—a compound linked to neurodegeneration and amyloid plaque formation—offering another avenue for AD prevention [171]. Regarding the mental health benefits of vegan and vegetarian diets, they may also indirectly reduce neurodegenerative risk. Regarding compounds like quercetin [20], found in plant foods, Zhang et al. demonstrated that this enhances mood-regulating neurotransmitters such as serotonin and dopamine, potentially alleviating anxiety and depression. Additionally, chronic stress and depression are known risk factors for AD, making these mental health improvements particularly relevant [172].

Caloric and fat reductions in vegan and vegetarian diets, alongside the exclusion of arachidonic acid (a pro-inflammatory compound in animal products), support the reduction in chronic inflammation [9,173]. For instance, a recent systematic review carried out by Barbaresko and collaborators found that cognitive health was boosted by reducing this dietary pattern, stopping systemic inflammation and promoting metabolic stability [10]. While these findings are promising, research gaps remain. Most studies are observational or based on small sample sizes, and more longitudinal and intervention studies are needed to establish causality. Additionally, although vegan diets are associated with lower inflammatory markers and healthier gut microbiota, results across studies are sometimes inconsistent [174].

In conclusion, vegan and vegetarian diets may act as both primary prevention—reducing neurodegenerative disease risk factors—and secondary prevention, mitigating disease progression through anti-inflammatory and neuroprotective mechanisms. However, diet diversity and proper nutrient planning are critical for optimizing these benefits. Further research is essential to fully understand the long-term impact on prevention and progression [155].

### 9.2. Detrimental Effects

Additionally, A vegan diet is limited in variety and offers fewer food choices, which raises the likelihood of developing deficiencies in certain nutrients [119]. Concerning micronutrient intake, two recent systematic reviews identified notable disparities between the nutrient profiles of meat-eaters and vegans. Vegan diets were linked to insufficient consumption of vitamins B2, B3, B12, and D, as well as iodine, calcium, selenium, and zinc [175,176]. This evidence may be linked to the fact that veganism or vegetarianism were associated with an increased risk of depression and lower anxiety levels [177], although no significant differences were observed in other outcomes. Iguacel and collaborators’ subgroups analyses indicated a heightened risk of anxiety, particularly among individuals under 26 years old and in studies of higher methodological quality. Further high-quality research is necessary to clarify these potential positive or negative relationships [121]. Overall, this suggests that there may be a potential link between veganism and an increased risk of anxiety, particularly in younger individuals and in studies of higher quality. However, the evidence is not conclusive, and more robust research is needed to better understand this relationship.

Focusing on these deficiencies, vitamin B12 plays a crucial role in maintaining the health of the brain and nervous system, particularly in the development and upkeep of the myelin sheath [177]. As a cofactor in essential enzymatic processes, B12 supports methionine synthesis, which is critical for proper myelin metabolism [178,179]. Deficiency in this vitamin can lead to neurological symptoms, including axonal demyelination and increased cerebral atrophy [180,181]. Elevated homocysteine levels are a reliable indicator of B12 deficiency and are associated with a higher risk of AD [140]. However, fortified foods and supplements effectively prevent these deficiencies, enabling vegans to maintain healthy B12 levels.

**Figure 4:**
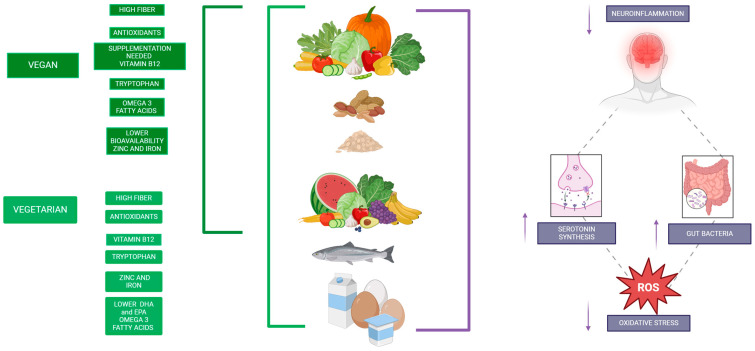
The beneficial and detrimental contributions of both diets to neurodegenerative conditions. These contributions are represented using dark green for a vegan diet and light green for a vegetarian diet.

Also, vitamin D deficiency is another concern associated with vegan diets, as plant-based sources of this vitamin are limited [182]. While sunlight exposure and fortified foods like plant milk and orange juice can help, supplementation with vitamin D2 or vegan-friendly D3 is often necessary [182]. Low vitamin D levels have been linked to increased risks of cognitive impairment, given the vitamin’s neuroprotective properties [183]. Additionally, omega-3 fatty acids [26], particularly eicosapentaenoic (EPA) and docosahexaenoic acids (DHA), are also scarce in vegan diets as they are primarily found in fish and seafood. Although α-linolenic (ALA), a plant-based omega-3 found in sources like flaxseeds and walnuts, can be converted to EPA and DHA, this conversion is inefficient [184]. Deficiencies in these fatty acids may impact brain health by reducing amyloid clearance and promoting inflammation, both of which are linked to cognitive decline, specifically in AD. While DHA supplementation shows benefits in early AD stages, it is less effective in advanced stages [185]. To address these concerns, the regular supplementation of B12, vitamin D, and omega-3s is highly recommended for vegans. However, more research is needed to evaluate the long-term effects and potential side effects of supplementation, such as the increased risk of prostate cancer associated with high omega-3 levels (**Figure 4**).

## 10. Benefits of Vegan and Vegetarian Diets

Vegan and vegetarian diets offer several potential benefits for neurological health, primarily due to their high content of phytonutrients, antioxidants, and anti-inflammatory compounds. These benefits are mediated through various mechanisms, including the reduction in oxidative stress, modulation of inflammation, and support for gut–brain axis interactions.

1. Reduction in Oxidative Stress and Neuroinflammation

Vegan and vegetarian diets are rich in bioactive compounds such as polyphenols, flavonoids, and carotenoids, which have been shown to play a crucial role in reducing oxidative stress and inflammation, two major contributors to neurodegenerative diseases such as Alzheimer’s and Parkinson’s. Studies have demonstrated that individuals adhering to plant-based diets exhibit lower levels of inflammatory biomarkers, such as C-reactive protein (CRP) and interleukin-6 (IL-6), suggesting a protective effect against neuroinflammation.

2. Support for Cognitive Function and Mood Regulation

The high intake of dietary fiber in vegan and vegetarian diets fosters a healthy gut microbiome, which, in turn, influences brain health through the gut–brain axis. Short-chain fatty acids (SCFAs) produced by gut bacteria, such as butyrate, have neuroprotective properties and are associated with reduced risks of depression and cognitive decline. Moreover, vegan and vegetarian diets provide essential precursors for neurotransmitter synthesis, such as tryptophan for serotonin production, which may contribute to improved mood and mental well-being.

3. Potential for Lowering the Risk of Neurodegenerative Diseases

Epidemiological studies suggest that vegan and vegetarian diets are associated with a lower risk of cognitive impairment and neurodegenerative diseases. The Mediterranean and MIND diets, which emphasize plant-based components, have been linked to a slower rate of cognitive decline and a reduced incidence of Alzheimer’s disease. While vegan and vegetarian diets require careful planning to avoid nutrient deficiencies, their neuroprotective effects are well documented when appropriately supplemented.

4. Cardiovascular Benefits and Cerebral Perfusion

A well-balanced vegan or vegetarian diet improves vascular health by reducing blood pressure, improving lipid profiles, and enhancing endothelial function. These cardiovascular benefits translate into improved cerebral blood flow, ensuring adequate oxygen and nutrient delivery to the brain, which is essential for cognitive preservation and mental clarity.

5. Neuroprotective Role of Specific Nutrients in Vegan and Vegetarian Diets
  - Polyphenols found in berries, green tea, and cocoa have been linked to improved cognitive performance and memory retention.
  - Flavonoids, particularly those in citrus fruits and dark chocolate, enhance neurogenesis and synaptic plasticity.
  - Carotenoids, such as lutein and zeaxanthin from leafy greens, support visual processing and cognitive resilience in aging populations.

While these diets present certain risks related to nutrient deficiencies (e.g., vitamin B12, DHA, iron), proper dietary planning, supplementation, and food fortification strategies can help mitigate these concerns, maximizing the neurological benefits of plant-based eating.

### Practical Applications

The findings of this review highlight key practical applications for optimizing the neurological benefits of vegan and vegetarian diets while addressing their associated risks. These strategies focus on supplementation, dietary planning, fortified foods, regular monitoring, and education to ensure these diets are nutritionally adequate and supportive of cognitive and overall neurological health (**Table 2**).

## 11. Conclusions

This review underscores the dual nature of vegan and vegetarian diets in neurological health. While rich in antioxidants and phytonutrients that reduce oxidative stress and inflammation, these diets may also introduce risks of nutrient deficiencies with significant implications for cognitive function and neurodegeneration. Addressing these challenges requires targeted dietary strategies including supplementation, food fortification, and preparation techniques that enhance nutrient bioavailability. By tailoring plant-based diets to individual needs and leveraging emerging technologies like biofortification and nutrigenomics, it is possible to maximize their neurological benefits while minimizing risks. Future longitudinal studies should further explore these interactions to refine dietary recommendations and improve outcomes across diverse populations.

## Figures and Tables

**Table 2:** Practical applications.

Category	Strategy	Key Recommendations
Supplementation	Vitamin B12	- Essential for all vegans/vegetarians (absent in unfortified plant-based foods). - Use supplements or fortified foods (plant-based milks, breakfast cereals, nutritional yeast) and regular monitoring to prevent deficiencies linked to cognitive decline, mood disorders, and neuropathy.
	Omega-3 Fatty Acids (DHA/EPA)	- Use algae-based supplements, the most effective and sustainable option, critical for maintaining neuronal integrity, synaptic plasticity, and reducing neuroinflammation.
	Iron and Zinc	- Non-heme iron and zinc from plant sources have lower bioavailability: pairing with vitamin C can enhance absorption. Iron supplementation is important for those at risk of anemia, while zinc supports immune function and cognitive processes.
Dietary Strategies	Emphasize Whole Nutrient-Dense Foods	- Focus on ALA-rich foods (flaxseeds, chia seeds, hemp seeds, walnuts) as precursors to omega-3 fatty acids. - Incorporate antioxidant-rich fruits and vegetables (e.g., berries, leafy greens) to combat oxidative stress and support neurological health.
	Improve Mineral Bioavailability	- Utilize methods such as soaking, sprouting, and fermenting to reduce antinutrients (e.g., phytates). - Pair iron-rich foods (e.g., legumes, tofu) with vitamin C-rich options (e.g., bell peppers, citrus fruits) to optimize nutrient uptake.
	Include Protein Variety	- Ensure a diverse intake of plant-based proteins (e.g., lentils, quinoa, soy products) to provide a complete amino acid profile and support neurotransmitter production.
Fortified Foods and Monitoring	Incorporate Fortified Products	- Regularly consume fortified foods containing B12, calcium, iodine, and other critical nutrients (e.g., fortified plant-based milks, cereals, iodized salt).
	Regular Biomarker Assessments	- Periodically test levels of key biomarkers (B12, DHA/EPA, iron, zinc). Monitor cognitive function (e.g., memory and executive processing) to identify early signs of nutrient deficiencies, particularly in high-risk groups (pregnant individuals, children, older adults).
Education and Awareness	Nutritional Guidance	- Provide comprehensive education on balanced vegan and vegetarian diets, including meal planning, proper supplementation, and food preparation techniques. - Offer tailored advice for specific populations (e.g., athletes, children, older adults) who may have heightened nutrient requirements.
	Community and Healthcare Involvement	- Encourage collaboration with healthcare providers to develop personalized dietary plans and ensure ongoing support for individuals transitioning to or maintaining plant-based diets.

## Data Availability

Not applicable.

## Key messages:

- Nutritional deficiencies in vegan and vegetarian diets, such as vitamin B12 and omega-3s, pose risks for cognitive function and neurological health.
- Antinutritional factors can impair nutrient absorption, but can be mitigated through preparation techniques like fermentation and sprouting.
- Vegan and vegetarian diets influence the gut–brain axis, emphasizing the importance of microbiota in cognitive and emotional health.
